# Using a reasoned action approach to identify determinants of organized exercise among Hispanics: a mixed-methods study

**DOI:** 10.1186/s12889-019-7527-1

**Published:** 2019-08-28

**Authors:** Mariana Arevalo, Louis D. Brown

**Affiliations:** 1 School of Public Health, Health Promotion and Behavior Sciences Department, The University of Texas Health Science Center at Houston, 7000 Fannin Street, #2502B, Houston, TX 77030 USA; 2 School of Public Health in El Paso, Health Promotion and Behavior Sciences Department, The University of Texas Health Science Center at Houston, 5130 Gateway East Blvd., MCA 316, El Paso, TX 79905 USA

**Keywords:** Elicitation, Latino, Attitudes, Subjective norms, Perceived behavioral control, Self-efficacy

## Abstract

**Background:**

Research on Hispanics’ activity preferences suggest that they prefer engaging in group-oriented physical activities, such as organized exercise. Yet, little is known about pathways to participation in organized exercise among Hispanics. This study used a reasoned action approach (RAA) framework to explore beliefs and determinants of organized exercise among Hispanics. Specifically, we examined the impact of participants’ intentions on reported organized exercise behavior, and the relation between intentions and attitudes, subjective norms, perceived behavioral control, and self-efficacy.

**Methods:**

Our mixed-methods study was part of a larger pre-post design intervention study. Participants completed an interview containing open- and closed-ended questions to identify salient beliefs and practices about attending organized exercise activities. We conducted two separate regression models to assess the effects of intentions on behavior (*n* = 330) and the associations of RAA constructs on intentions (*n* = 101), both adjusting for demographics. Qualitative analysis of a sub-sample (*n* = 105) of responses to open-ended questions identified salient beliefs related to organized exercise attendance.

**Results:**

Our results showed that intentions predicted behavior at follow up (IRR = 2.03, *p* < .05), and that attitudes and perceived behavioral control were associated with intentions (β = .36, *p* < .05; β = .36, *p* <. 05, respectively). Qualitative findings suggest participants value health and the behavioral benefits of attending organized exercise activities; feel approval from family and friends; and identify transportation, time, distance, and costs as factors that influence their attendance to organized exercise activities.

**Conclusions:**

Consistent with theoretical expectations, we identified statistically significant determinants of intentions and attendance to organized exercise. Findings can inform the development of persuasive messages and interventions to promote exercise in low-income Hispanic populations facing obesity disparities.

**Electronic supplementary material:**

The online version of this article (10.1186/s12889-019-7527-1) contains supplementary material, which is available to authorized users.

## Background

Despite the many documented benefits of exercise, Hispanics continue to be less physically active than their White counterparts [1]. It is estimated that only 25% of Hispanics meet leisure-time physical activity guidelines, compared to 35% of non-Hispanic Whites; and only 15% of Hispanics meet muscle-strengthening guidelines compared to 24% of their counterparts [2]. Researchers have found that Hispanics prefer engaging in group-oriented physical activities such as dancing, walking, gardening, and family- and peer-oriented activities [3, 4]. One type of group-oriented physical activity is organized exercise, which includes planned or structured group activities, classes or lessons, and team sports performed to improve or maintain physical fitness [5]. To help promote physical activity in Hispanics, it is important to study determinants and pathways to participation in organized exercise.

Researchers have used various health behavior theories to understand personal, behavioral, and environmental factors related to physical activity and exercise behavior. One commonly used theoretical framework to study exercise behavior is a reasoned action approach (RAA) [6]. The RAA umbrella includes Theory of Reasoned Action (TRA [7];), Theory of Planned Behavior (TPB [8];), and the Integrative Behavior Model (IBM [9];). The RAA framework is a particularly relevant perspective to study exercise because of the planned or reasoned aspect of exercise behavior [10]. This framework proposes that intentions to engage in a behavior are the most immediate determinant of that behavior. In turn, intentions are influenced by attitudes, normative pressure, self-efficacy, and perceived behavioral control; and each of these constructs is formed by salient behavioral, normative, and control beliefs that should be elicited from the target population [6, 11, 12].

Evidence about the application of these behavioral theories to study exercise conclude that the most influential constructs to intentions are attitudes and perceived behavioral control [13, 14]. A meta-analysis of 162 studies using TRA or TPB found large effect sizes (ES) between attitudes and intentions (ES = 1.22, SD = 0.05) and perceived behavioral control and intentions (ES = 0.97, SD = 0.04), but moderate effect sizes between subjective norms and intentions (ES = 0.56, SD = 0.07) [13]. Authors also found a large effect size between intentions and exercise behavior (ES = 1.04, SD = 0.08). Their findings are consistent with a more recent meta-analysis of 72 studies exploring the application of TRA and TPB on physical activity [14]. Additionally, authors found that self-efficacy and past behavior had a statistically significant influence on intentions, and thus are important constructs to study. For this reason, our study used an extended version of TPB which incorporated self-efficacy as a predictor of intentions, still under the guidance of a RAA framework.

Although many researchers have used theories under the RAA umbrella to explore determinants of exercise behavior [15], to the best of our knowledge, beliefs and practices about *organized exercise* have not yet been studied using a RAA framework. Researchers have found differences in determinants of physical activity and exercise among people of diverse ethnic and racial groups [16, 17], which lead us to believe that there may also be differences in beliefs and practices of organized exercise among Hispanics. This is an important area to study because many interventions targeted to Hispanics include organized exercise activities such as dance classes, walking clubs, and others group-based exercise activities conducted in a variety of settings including community and faith organizations [17–19].

Our study aimed to identify salient beliefs and practices about attending organized exercise activities among cohort participants in *Healthy Fit*, a program which used community health workers to link community members to services and resources addressing Hispanic health disparities. We used a mixed-methods study approach. First, we qualitatively explored participants’ perceived benefits and negative aspects of attending organized exercise activities using an elicitation technique, which help explore underlying cognitive structures of beliefs [11]. Then, we quantitatively examined the associations of participants’ attitudes, perceived norms, self-efficacy, and perceived behavioral control on their intentions to attend organized exercise activities, and examined the influence of intentions on attendance to organized exercise activities at 30-day follow up. Based on the RAA framework, we hypothesized that attendance to organized exercise activities would be predicted by intentions, and that attitudes, subjective norms, self-efficacy, and perceived behavioral control would be associated with intentions.

## Methods

This is a mixed-methods study, which is part of a larger pre-post design intervention study, called *Healthy Fit* [20]. Bilingual study staff recruited men and women from local health fairs, Mexican consulate’s *Ventanilla de Salud* (health window, in English), housing complexes, and community centers in El Paso, TX and its vicinity areas. Individuals were eligible to participate in the *Healthy Fit* study if they were over the age of 18 years, male or female, and not pregnant. Community health workers (CHWs) conducted 20-to-30-min interviews with each participant, in Spanish or English, to assess different health needs. Depending on the participants’ responses and study eligibility guidelines, CHWs provided referrals to community resources, including free organized exercise classes and other local physical activity programs. CHWs interviewed participants at baseline and 30 days after enrollment (i.e. follow-up). CHWs randomly selected a subsample of *Healthy Fit* participants to complete an additional questionnaire assessing RAA constructs. CHWs obtained informed consent from all participants. The UTHealth Institutional Review Board (IRB) reviewed and approved this study.

**Fig. 1:**
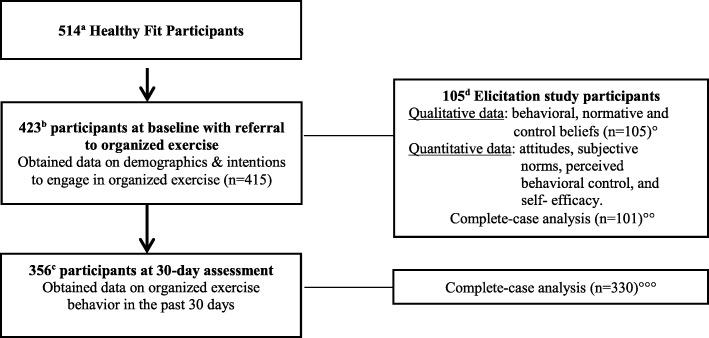
Flow chart of study participation and analysis shows **a** number of participants in the larger study. **b** number of participants who received a referral to organized exercise. **c** number of participants at 30-day follow up who answered questions about attendance to organized exercise. **d** number of participants who completed the elicitation questionnaire. ° denotes analytical sample for qualitative analysis. °° denotes analytical sample for model examining association between intentions and RAA constructs. °°° denotes analytical sample for model assessing impact of intentions on behavior.

Baseline data were collected between 2015 and 2016. During this period, a total of 514 people participated in the *Healthy Fit* study (see Fig. 1). Of those, 423 (82.29%) were overweight or had blood pressure measures above normal levels, and thus received an organized exercise referral. Among participants receiving an organized exercise referral, 415 (98.10%) participants reported their intentions to attend organized exercise at baseline; at one-month follow up, 356 (85.78%) participants reported their past 30-day attendance to organized exercise activities. From those who received an organized exercise referral, a randomly selected sub-sample of 105 individuals were asked to complete an organized exercise elicitation questionnaire, described in more detail below.

### Measures

At baseline, all participants completed a socio-demographic survey, including questions regarding their gender, age, marital status, employment status, annual household income, insurance status, and education. Body Mass Index (BMI) and blood pressure were also assessed. Participants who were overweight (i.e., BMI ≥ 25 kg/m^2^) or had blood pressure levels above normal (i.e., systolic mm Hg ≥ 120 or diastolic mm Hg ≥ 80) received a referral to community-based organized exercise and were asked about their intentions to attend organized exercise activities in the next 30 days. We defined intentions as an individual’s readiness to perform the behavior by combining three items: 1) level of motivation to attend organized exercise activities in the next 30 days, measured on a 7-point scale (e.g. *completely unmotivated – completely motivated*); 2) level of determination to attend organized exercise activities in the next 30 days, measured on a 7-point scale (e.g. *completely undetermined – very determined*); and 3) number of days participants planned to attend organized exercise activities in the next 30 days. We standardized each variable by calculating z-scores, and subsequently computed the mean of those z-scores into a single, standardized measure of intentions (α = .84).

A sub-sample of our baseline participants who received an organized exercise referral were asked to complete an additional elicitation questionnaire. This questionnaire contained open- and closed-ended questions assessing RAA constructs, and we developed it following theoretical recommendations on how to construct TPB measures [11, 21]. One section of the questionnaire utilized an elicitation technique to obtain participants’ salient beliefs that might influence attendance to organized exercise activities. This section consisted of eight open-ended questions, such as: 1) *how do you feel about the idea of going to organized exercise activities? 2) what are the plusses of you going to exercise activities in the next 30 days?* (See Additional file 1 for elicitation questions in English and Spanish language). Another section of the questionnaire quantitatively assessed RAA constructs: attitudes, subjective norms, perceived behavioral control, and self-efficacy.

Attitudes, defined as an emotional response about performing the behavior and beliefs about the outcome of the behavior, were assessed with 6 items corresponding to responses to the stem: “going to organized exercise activities in the next 30 days would be…” The response scales ranged from 1 to 7 points (e.g.*, very unpleasant – very pleasant; very boring – very exciting; very harmful – very beneficial;* α = .94). Subjective norms, referring to participants’ normative beliefs about their perceived expectations of others about what they should do, were assessed by 3 items. A sample item is *“Most people who are important to me would want me to go to exercise activities.”* The response scale ranged from 1 to 7 (e.g.*, completely untrue – completely true;* α = .81). Perceived behavioral control, referring to an individuals’ perceived amount of control over the behavior, was assessed by 3 items (α = .88). A sample item is: *“I have complete personal control over going to organized exercise activities if I really wanted to do so.”* The response scale ranged from 1 to 7 (e.g.*, strongly disagree – strongly agree*). Self-efficacy, or a participant’s perceived capability to perform the behavior, was assessed by 5 items corresponding to responses to the stem: “*I can manage to carry out my intentions to go to organized exercise activities in the next 30 days.”* The response scale ranged from 1 to 7 (e.g., very unsure – very sure; α = .96).

At follow up, 30 days after enrollment, participants were asked to complete another questionnaire administered by the CHWs. Our primary outcome of interest was assessed by asking participants the number of organized exercise activities they had attended in the last 30 days. Participants were given the choice to report attendance to two different activities in the past 30 days, and were asked to report the number of times per week they attended each activity. We combined these two activities into a single variable to reflect the number of times the participant attended organized exercise activities in the last 30 days. Also, at follow up, participants were asked open-ended questions about what they had liked and disliked about attending these organized exercise activities.

### Data analysis

#### Qualitative analysis

Following data collection, the first author categorized all open-ended questions by hand [11]. Following theoretical recommendation for conducting analysis on elicitation study data, she tabulated frequency counts of all responses and classified them by a-priori themes based on belief type: behavioral, normative, and control. The most frequent responses were subsequently used to create items assessing indirect measures of RAA constructs, such as salient behavioral beliefs, outcomes evaluations, salient normative beliefs, motivations to comply, salient control beliefs and perceived control (See Additional file 2).

#### Quantitative analysis

We coded variables as follows: gender (1 = female, 0 = male), age, marital status (1 = married, 0 = not married), employment status (1 = employed, 0 = unemployed), annual household income (1 = 0–19,999, 2 = 20,000 – 29,999, 3 = 30,000+), insurance status (1 = insured, 0 = uninsured), and education (1 = less than high school, 2 = High school or GED, 3 = Some college or Associate’s degree, 4 = College Graduate or higher degree). Additionally, we computed scale scores for each RAA measure by calculating the average of non-missing scores for the 6 items assessing attitudes, 3 items assessing subjective norms, 3 items assessing perceived behavioral control, 5 items assessing self-efficacy, and 3 items assessing intentions, as described above. Exercise behavior was a count variable reflecting the number of times the participant attended organized exercise activities in the last 30 days.

We conducted quantitative analyses using Stata version 13 [22]. First, we conducted descriptive statistics to assess the distribution of the variables, examined means, standard deviations, and frequencies. Then, we used Pearson’s correlation coefficients to determine the bivariate associations between all RAA variables and behavior. We also calculated Cronbach’s alpha to assess the internal consistency reliability of our RAA measures. Next, we estimated the range of energy expenditure of the activities reported by participants using the Compendium of Physical Activity [23].

Lastly, we conducted two separate regression models to identify determinants of organized exercise in our sample. First, we used a multiple regression model to examine the association between the RAA constructs and intentions, while adjusting for demographic variables. Next, we examined the impact of intentions on behavior, while adjusting for demographic variables. We used a negative binomial model because our outcome variable was over-dispersed [24]. We used a forward stepwise procedure to select potential socio-demographic covariates. To select the best subset of predictors, a significance level for removal from the model was set at α < 0.20. For both models, we used a complete-case analysis approach for handling missing data. In the first model predicting intentions, of the 105 participants selected for RAA questions on organized exercise, we have complete data on 101 (96.19%). In the second model predicting attendance, among the 356 participants with follow-up data, we have complete data on 330 (92.69%).

## Results

Table 1 presents demographic characteristics for participants in the elicitation portion and survey portion of the study. The majority of participants self-identified as Hispanic/Latino, were female, married, not employed, with a household annual income of less than $19,999, with less than a high school diploma, uninsured, had a BMI average of about 30 (SD = 5.5), and a mean age of 45 ± 13 years. Participants reported attending a variety of organized exercise activities ranging from Zumba to sports like volleyball, soccer, and boxing. The most commonly reported activity attended was Zumba, estimated at 7.5 metabolic equivalents (METs).

**Table 1:** Participant demographic characteristics

Characteristic	Elicitation portion 105 N (%)	Survey portion 330 N (%)
Age (mean, SD)	44.92 (13.51)	47.03 (12.89)
Range	18–79	18–76
BMI (mean, SD)	31.89 (5.54)	32.03 (5.59)
Range	23–52	19–52
Ethnicity		
Hispanic/Latino	100 (96.15)	322 (97.58)
Gender		
Female	81 (77.88)	268 (81.21)
Male	23 (22.12)	62 (18.79)
Marital Status		
Currently married	56 (53.85)	162 (49.09)
Divorced	9 (8.65)	33 (10.00)
Widowed	5 (4.81)	19 (5.76)
Separated	10 (9.62)	35 (10.61)
Never Married	20 (19.23)	64 (19.39)
Living together as couple	4 (3.85)	14 (4.24)
Employment status		
Employed	40 (38.46)	116 (35.15)
Not employed	63 (60.58)	214 (64.85)
Insurance Status		
Not insured	79 (75.96)	267 (80.91)
Insured	24 (23.08)	62 (18.79)
Annual Household Income		
Less than $19,999	69 (66.35)	233 (70.61)
$20,000–$29,999	20 (19.23)	56 (16.97)
$30,000 or more	14 (13.46)	37 (11.21)
Education level		
Less than high school diploma	39 (37.50)	130 (39.39)
High school graduate or equivalent	35 (33.65)	111 (33.64)
Some college or associate’s degree	22 (21.15)	63 (19.09)
Bachelor’s degree or higher	6 (5.77)	26 (7.88)

### Beliefs about organized exercise behavior

We present our findings on beliefs about organized exercise behavior by the following themes: behavioral advantages and disadvantages, normative referents for and referents againsts, and control beliefs – see Table 2. The most salient behavioral advantages reported by participants were that it improves physical health, aids weight loss, and improves motivation to stay fit. The most common reported behavioral disadvantage was time constraints. The most salient normative referents in support of participants engaging in organized exercise activities were family members, including children and spouses. Most participants reported having no referents against engaging in the behavior, but some reported family members. The most salient control beliefs reported by our participants as barriers to attendance to organized exercise activities included lack of transportation, lack of time, distance, and having no money to pay for gas, parking, or gym fees. The most salient reported facilitators were having free time, having a car, close proximity to activities, having money to pay for fees, and having support from their family.

**Table 2:** Most frequently reported behavioral, normative, and control beliefs about attending organized exercise activities

Theme	Response	Count*
Behavioral beliefs - Advantages of behavior		
Improves psychological health	Improves motivation to stay fit	17
	Improves mood (feeling excited, good about oneself)	9
Improves physical health	Improves physical health	36
	Helps get or stay in shape	7
Weight control	Helps to lose weight	24
Improves social life	To meet new people	8
Increases energy	Increases energy	6
Behavioral beliefs - Disadvantages of behavior		
Constraints	Lack of time	9
None	No disadvantages reported	51
Normative beliefs - Referents in support of behavior		
Family	Children	30
	Spouse or significant other	27
	Family in general	20
Others	Friends	9
Normative beliefs - Referents against behavior		
Family	Family members	5
Control beliefs - Barriers		
Lack of transportation	No car or transportation	16
Time	Lack of time	9
Lack of convenience	Long Distance or far location	8
Money	No money for gas, gym fees, parking fees	7
Control beliefs - Facilitators		
Behavioral	Being motivated	11
Convenience	Having free time	17
	Distance/location (close to home)	9
	Price/cost	5
Having transportation	Having a car	9
Perceived social support	Support from family	6

*counts do not add up to 105 because these responses are only the most frequently reported by participants

### Inter-correlations between main variables

Bivariate correlations between the RAA constructs were positive and significant (Table 3). Inter-correlations between RAA constructs and attendance to organized exercise were all small, and not statistically significant. Inter-correlations between RAA constructs and intentions were all medium to large in magnitude and statistically significant. The largest correlations were between attitudes and perceived behavioral control (*r* = .75, *p* < .001), and subjective norms and perceived behavioral control (*r* = .75, *p* < .001).

**Table 3:** Descriptive statistics and correlations between Reasoned Action Approach constructs and behavior (N = 101)

Variable	2	3	4	5	6	M	SD	α
1. Behavior	.14*	.12	.01	.07	.10	2.13	5.77	–
2. Intentions		.66**	.46**	.63**	.54**	0.00	1.00	.84
3. Attitudes			.69**	.75**	.68**	5.75	1.27	.94
4. Subjective Norms				.75**	.43**	6.04	.94	.84
5. Perceived Control					.60**	5.74	1.24	.90
6. Self-efficacy						4.90	1.66	.96

*p < .05; **p < .001

*M* = mean; *SD* = standard deviation; ***α*** **=** Cronbach’s alpha

### Predicting intentions to attend organized exercise activities

Results from the multiple regression model indicated that intentions were statistically associated with attitudes and perceived behavioral control, while adjusting for gender, education, and other RAA variables in the model (β = .36, *p* < .05, 95% C.I.: .20 to .52; β = .36, *p* < .05, 95% C.I.: .20 to .53, respectively). We calculated the standardized beta coefficients to compare the strengths of coefficients to other variable coefficients in the model. Results indicated that one standard deviation increase in attitudes yielded a .36 standard deviation increase in intentions; whereas one standard deviation increase in perceived behavioral control yielded a .36 standard deviation increase in intentions. Subjective norms and self-efficacy were not significantly associated with intentions (β = −.13, *p* = 0.26, 95% C.I.: − 0.40 to 0.13; β = .07, *p* = 0.46, 95% C.I.: 0.00 to 0.15, respectively). See Table 4.

**Table 4:** Model assessing association between intentions to attend organized exercise activities and Reasoned Action Approach constructs, adjusting for gender and education (N = 101)

Indicator	b	95% CI	β	95% CI	*p*-value
Attitudes	0.28	0.07–0.48	0.36	0.20–0.52	0.00
Subjective norms	−0.14	−0.39-0.11	−0.13	−0.40-0.13	0.26
Perceived behavioral control	0.29	0.08–0.50	0.36	0.20–0.53	0.00
Self-efficacy	0.04	−0.07-0.16	0.07	0.00–0.15	0.46
Gender					
Male	−.015	−0.54-0.22	−0.06	− 0.96-0.83	0.41
Female	Ref	–	–	–	–
Education level					
High school	0.22	−0.11-0.56	0.11	−0.24-0.46	0.19
Some college	0.21	−0.17-0.60	0.08	−0.32-0.50	0.28
College degree+	0.20	−0.42-0.83	0.05	0.61–0.71	0.52
Less than high school diploma	Ref	–	–		–
R^2^ = 0.49					
Adj R^2^ = 0.45					

*b* = regression coefficients; *CI* = Confidence Interval; *β* = standardized regression coefficients

### Predicting behavior on intentions

Results of the negative binomial regression model (see Table 5) indicated that intentions predicted attendance to organized exercise behavior at follow up (IRR = 2.03, *p* < .05, CI: 1.10 to 3.76), while controlling for gender, education, and age. Thus, a one standard deviation increase in intentions predicted attendance at twice as many organized exercise activities in the past 30 days.

**Table 5:** Model predicting attendance to organized exercise activities on intentions, adjusting for gender, age, and education level (N = 330)

Predictor	IRR	95% CI	p-value
Intentions	2.03	1.10–3.76	0.02
Gender			
Male	0.08	0.01–0.47	0.00
Female	Ref	–	–
Age	0.95	0.91–1.00	0.05
Education level			
High school	1.18	0.37–3.69	0.77
Some college	4.61	0.76–28.03	0.09
College degree+	1.86	0.29–11.95	0.51
Less than high school diploma	Ref	–	–

*IRR* = Incident rate ratio; *CI* = Confidence Interval

Overall, 16% of participants referred to attend organized exercise activities reported attendance at the 30-day follow-up interview, compared to 84% who reported no attendance. Those who reported attending organized exercise activities in the past month said they had attended an average of 2 times per week. When asked what they liked about attending to organized exercise activities in their community, many participants reported that it made them feel good, energized, relaxed, helped improve or maintain their health, lose weight, and meet new people. Also, many participants said that they liked going to Zumba classes. When asked about what they disliked about going to organized exercise activities, most people said “nothing,” and a few participants said that they disliked the pain or discomfort after exercising.

## Discussion

The RAA framework helped us identify determinants of attendance to organized exercise in a predominantly low-income Hispanic sample on the US-Mexico border. To our knowledge, no other study had examined the applicability of the RAA framework to predict attendance to organized exercise behavior. As theoretically expected, our findings indicated that participants’ intentions to perform organized exercise activities predicted subsequent attendance to these activities. We also found that attitudes and perceived behavioral control were significant correlates of intentions, highlighting the importance of participant’s affective and cognitive beliefs, and perceived control over the behavior in the formulation of behavioral intentions. These findings are consistent with another study that found attitudes and perceived behavioral control as significant correlates of intentions [25]. These findings can help researchers develop persuasive messages to promote participation, and design strategies targeting factors that would facilitate engagement in organized exercise.

Our results also indicated that subjective norms and self-efficacy had a relatively small and non-significant association with intentions. This is consistent with findings from other researchers studying Hispanic subpopulations [26]. After examining constructs from health behavior theories in racial- and ethnically diverse populations, Burke and colleagues [27] found that norms develop in the context of relational culture, meaning that the formulation of norms depend on the level of connectedness among referent individuals. Our three-item measure of subjective norms included only one item assessing descriptive norms, and it did not ask about specific referents that were deeply connected to our participants. Perhaps further refinement of our measure would yield an association with intentions.

Our qualitative elicitation findings provided valuable insights into the salient beliefs related to organized exercise in a predominantly low-income Hispanic sample. Results shed light on participants’ values about attending organized exercise activities, identified key members within their personal networks that could help promote or reinforce engagement in exercise, and drew attention to a variety of logistical barriers that may impede their participation in organized exercise. For example, individuals’ control beliefs about transportation suggest exercise activities need to be widely available in the community so that they are close to the participants’ homes. Further, participants’ behavioral beliefs suggest appeals to psychological and physical health may be effective motivators for participation. Finally, participant’s normative beliefs on the importance of family in supporting exercise suggest family-centered interventions may be particularly effective. One study that explored preferences and needs related to leisure-time physical activity among Mexican-Americans found similar results about barriers to participation including lack of motivation, time, money and transportation [4]. However, they found a lack of social support from partners/husbands and friends, whereas our sample reported having family social support and most of them did not name any referents against engaging in organized exercise [4]. We believe that our findings are particularly relevant since, to date, no other study had captured behavioral, normative and control beliefs regarding organized exercise for Hispanic communities of low socioeconomic status facing obesity disparities.

Elicitation findings helped us understand our quantitative findings. Our quantitative measure of self-efficacy assessed mood and affective barriers such as feeling tired, busy, being worried or depressed; however, our elicitation findings suggest some unassessed logistical barriers were relevant for our population including transportation, distance, and costs. This may explain why we did not find self-efficacy to be significantly associated with intentions. Thus, the elicited beliefs could help develop precise direct and indirect measures of RAA constructs for our population, and potentially contribute to the field measures of RAA constructs specifically developed from beliefs of ethnically diverse populations. In Additional file 2, we have included a list of items that we developed based on these findings, that we hope to use in future research.

### Strengths and limitations

Our study has a number of strengths and some important limitations. A major strength is our examination of culturally-relevant behavioral determinants guided by a RAA theoretical perspective. Given that Hispanics prefer engaging in group-oriented physical activities [3], our study offers valuable insights into the relevant beliefs and determinants of engagement in organized exercise. Also, our study can add to the body of knowledge on the applicability of the RAA framework in assessing pathways to organized exercise in Hispanics subpopulations. More work is still needed in this area, and future research should further explore the applicability of the RAA framework and behavioral mechanisms among diverse populations.

One study limitation was our modest sample size, largely comprised of females, which can limit the generalizability of our findings. To substantiate the generalizability of our findings, future studies should examine the utility of the RAA framework with a larger and more diverse sample, possibly with other Hispanic subpopulations. Another limitation had to do with our measure of intentions as a combined standardized score, which may make interpretation of findings related to intentions more difficult. However, we believe that our combined intentions score has good precision and is a better measure of intentions than using any of the three single items alone. Also, our study did not examine religious and other social ties that could be important variables to study in future research. Lastly, our data were self-reported, and it is possible that a social desirability bias may have led to inaccurate reports of intentions to attend or actual attendance to organized exercise activities.

### Recommendations for practice

Our findings regarding attitudes and perceived behavioral control are consistent with the notion that formulation of intentions to engage in exercise relies more on personal motivational judgements and perceived external aspects of control, and it is less influenced by normative pressure or one’s perceived ability to perform the behavior. For this reason, future interventions promoting participation in organized exercise should develop persuasive motivational messages and individual action plans that incorporate relevant behavioral beliefs, as it is likely that these beliefs would help formulate strong intentions, which would then translate to engagement in organized exercise. Interventions should also foster internal perceptions of control by encouraging organized exercise activities that individuals feel competent performing, but most importantly, incorporate strategies to address external barriers that might hamper participation.

## Conclusion

Understanding Hispanics’ beliefs about organized exercise activities can help researchers develop interventions and messages that address culture-specific beliefs related to organized exercise behavior. Our study offered an opportunity to assess the applicability of the RAA framework in an ethnically diverse population, provided evidence about the utility of value-expectancy constructs, and yielded a unique set of beliefs that our sample population holds regarding engagement in organized exercise. In this study we also developed RAA measures derived from these beliefs, which provide the groundwork for future measurement development studies. Such research can continue to advance current health behavior theoretical frameworks across populations and settings, inform exercise promotion efforts, and address Hispanic health disparities.

## Additional files

Additional file 1: Contains the elicitation open-ended questions, in English and Spanish language. (DOCX 17 kb)

Additional file 2: Contains the items developed based on our elicitation study findings, but not tested in our study. (DOCX 16 kb)

## Abbreviations

BMI: Body Mass Index
CHW: Community Health Workers
IBM: Integrated Behavioral Model
IRR: Incident Rate Ratio
MET: Metabolic Equivalent Tasks
RAA: Reasoned Action Approach
TPB: Theory of Planned Behavior
TRA: Theory of Reasoned Action
