# Supplementary material for: Using a reasoned action approach to identify determinants of organized exercise among Hispanics: a mixed-methods study
Source: BMC Public Health. 2019 Aug 28;19:1181. doi: 10.1186/s12889-019-7527-1 (PMC6714086; doi:10.1186/s12889-019-7527-1)
Supplement: Supplementary file 1 — Contains the elicitation open-ended questions, in English and Spanish language. (DOCX 17 kb) [file 12889_2019_7527_MOESM1_ESM.docx]

**Additional Files**

**Additional file 1**. Elicitation questions in English and Spanish language

*Behavioral beliefs*

1. How do you feel about the idea of going to organized exercise activities?
   1. (Probe if needed) What do you like about going to organized exercise activities?
   2. (Probe if needed) What do you dislike about going to organized exercise activities?
2. What are the plusses of you going to organized exercise activities?
3. What are the minuses of you going to organized exercise activities?

*Normative beliefs*

1. Who would support you going to organized exercise activities?
2. Who would be against you going to organized exercise activities?

*Control beliefs*

1. What things make it easy for you to go to organized exercise activities?
2. What things make it hard for you to go to organized exercise activities?

***SPANISH version***

*Behavioral beliefs*

1. ¿Cómo se siente acerca de la idea de asistir a actividades de ejercicio organizado?
   1. (aclare si es necesario) ¿Qué le gusta de asistir a actividades de ejercicio organizado?
   2. (aclare si es necesario) ¿Qué no le gusta de asistir a actividades de ejercicio organizado?
2. ¿Cuáles son las ventajas de asistir a actividades de ejercicio organizado?
3. ¿Cuáles son las desventajas de asistir a actividades de ejercicio organizado?

*Normative beliefs*

1. ¿Quién le apoyaría si decidiera asistir a actividades de ejercicio organizado?
2. ¿Quién podría estar en contra de que usted asista a actividades de ejercicio organizado?

*Control beliefs*

1. ¿Qué cosas hacen que sea fácil para que usted pueda asistir a actividades de ejercicio organizado?
2. ¿Qué cosas hacen que sea difícil para que usted pueda asistir a actividades de ejercicio organizado?
