# Supplementary material for: Using a reasoned action approach to identify determinants of organized exercise among Hispanics: a mixed-methods study
Source: BMC Public Health. 2019 Aug 28;19:1181. doi: 10.1186/s12889-019-7527-1 (PMC6714086; doi:10.1186/s12889-019-7527-1)
Supplement: Supplementary file 2 — Contains the items developed based on our elicitation study findings, but not tested in our study. (DOCX 16 kb) [file 12889_2019_7527_MOESM2_ESM.docx]

**Additional file 2.** Items developed based on our elicitation study findings, but not tested.

**BEHAVIORAL BELIEFS**

**Salient behavioral beliefs:** (7-point scale: extremely likely – extremely unlikely)

1. Participating in organized exercise activities would **improve my mood**
2. Participating in organized exercise activities would **keep me motivated to stay fit**
3. Participating in organized exercise activities would **improve my physical health**
4. Participating in organized exercise activities would **help me lose weight**
5. Participating in organized exercise activities would **help me stay in shape**
6. Participating in organized exercise activities would **increase my energy**
7. Participating in organized exercise activities would **help me meet new people**

**Outcome evaluations:** (7-point scale: extremely important – extremely unimportant)

*How important are each of the following for you?*

1. Having a good mood
2. Feeling motivated
3. Improving your health
4. Losing weight
5. Staying in shape
6. Increasing your energy levels
7. Meeting new people

**NORMATIVE BELIEFS**

**Salient normative beliefs:** (Strongly approve – Strongly disapprove)

How strongly would each of the following persons **approve** or **disapprove** of you participating in organized exercise activities?

1. Spouse, partner or significant other
2. Children
3. Siblings
4. Parents
5. Friends
6. Co-workers
7. School mates
8. Your doctor

**Motivation to comply:** (Strongly important – Strongly unimportant)

How important is it for you to do what your __________ approve(s) of?

[1-8 are contingent on previous question]

1. Spouse, partner or significant other
2. Children
3. Siblings
4. Parents
5. Friends
6. Co-workers
7. School mates
8. doctor

**PERCEIVED BEHAVIORAL CONTROL**

**Salient control beliefs: (**7-point scale: extremely weak – extremely strong)

How strongly would each of the following items influence your participation in organized exercise activities?

1. Distance or location
2. Time to attend
3. Program or schedule
4. Time off work
5. Access to transportation (car, bus, or carpooling)
6. Child care
7. Costs
8. Motivation
9. Willingness
10. Information about opportunities to exercise
11. Support from family
12. Support from friends

**Perceived control:** (0-10 scale: no control – a lot of control)

How much control do you have over participating in organized exercise activities even if…

1. It is far away from where you live
2. You do not have time to attend
3. The class schedule is not flexible
4. You cannot take time off work
5. You do not have access to transportation
6. You do not have someone to watch your children
7. You cannot afford it
8. You do not have the motivation to attend
9. You are not willing to go
10. You do not have information about organized exercise opportunities
11. You do not have support from your family
12. You do not have support from your friends
